# Chronic Perforating Ulcer of the Bladder

**Published:** 1907-08

**Authors:** Henry K. Leake

**Affiliations:** Dallas, Texas


### Chroni Perforating Ulcer of the Bladder.

BY HENRY K. LEAKE, A. M., M. D., DALLAS, TEXAS.

In the *Journal of the American Medical Association* of March 30, 1907, Dr. G. Walker gives a report of two personal observations of patients asserted to have have simple ulcer of the bladder. He describes two types of the disease—acute and chronic—and states that the symptomatology closely simulates that of cystitis, from which it can be differentiated only by cystoscopic examination.

Many years ago, in an article which I published on this subject, I said that in English literature Lawson Tait was the first to call the attention of the profession to this rare affection of the diseases of women, and that he had given the credit to Rokitansky for having preceded him in describing it. Writing to the *Lancet* for March, 1871, Tait gave the brief histories of four cases of chronic perforating ulcer of the bladder of which he had personal knowledge. Two of these cases he treated in association with his great master—Sir James Y. Simpson. The third case was in his individual practice and he was present at the post-mortem examination of a fourth case which, he says, put him in a position to prove the views of Rokitansky, the well known distinguished pathologist.

On analysis of Tait's article it is evident that in the first three cases he may have made an error in diagnosis, for the symptoms were not different from those of ordinary cystitis—a disease not infrequent in women—and as cystoscopy was then in an inchoate stage, no inspection of the bladder was undertaken. In the fourth case, I assume that no diagnosis was made previously to the death of the patient, after which the post-mortem discovered "a limited perforating ulcer," presumably situated at the neck of the bladder, for Tait says the perforating ulcer most frequently seems to exist at this point.

Tait had a double purpose in sending his communication to the *Lancet:* First, he eulogizes his revered teacher, Sir James Y. Simpson, for having originated the operation of vesicovaginal fistula to cure chronic perforating ulcer of the bladder; and, second, to warn the profession of the existence of a rare disease in women which, imitating cystitis in its clinical history, might deceive the physician who, relying upon the ordinary means for treating cystitis, might delay or neglect the adoption of surgical measures, by which alone the successful treatment of what Tait calls this "terrible disease," may be accomplished.

That, exceptionally, it may be, a perforating ulcer may exist in the bladder remote from its neck, I had occasion to show in the article above referred to, wherein I narrated the history of a very interesting and instructive case that had occurred in Dallas several years after the publication of Tait's article.

The wife of a prominent and greatly esteemed physician, recently deceased, a middle-aged and nervous woman but otherwise in the very best health, was taken with symptoms ordinarily attending inflammation of the bladder. Her condition growing gradually worse, several physicians were called in to the case. One of these urgently advised and ultimately made forcible dilatation of the urethra, the patient being anesthetized. The operation was followed by an aggravation of all the symptoms, which became progressively intensified to such a degree as to render the condition of the patient most pitiable. For weeks she suffered the greatest agony, except when under the influence of powerful opiates, which added their baneful effects to those of the original disorder. She was transferred to New York and placed under the care of a gynecologist of world-wide fame. From reports received I understood that this gynecologist was puzzled as to the exact nature of the case, but treated it on the hypothesis that the symptoms were owing to a severe form of cystitis, although he refrained from making a vesicovaginal fistula which had been recommended by Emmet in the treatment of such cases. The patient remained in New York several months without the slightest benefit from her visit, her condition from day to day becoming more serious. Finally she returned to Dallas and I was called to attend her. From a woman of good physique and lively temperament I found her emaciated, neurasthenic, and melancholy. With a view of at least giving rest to the bladder from its frequent and painful contractions, I made a free vesicovaginal fistula and, as suggested by Emmet, introduced a drainage button to keep the incision open. This procedure gave much immediate relief, but did not entirely remove the pain, which soon began and continued above the bladder. After weeks of constant suffering and exhaustion, death occurred, the patient retaining her mental faculties to the very last. A post-mortem examination disclosed a uniformly round ulcer in the region of the fundus of the bladder above the trigone, which had perforated all the coats. The edge of the ulcer was clean cut without undermining and with little if any thickening in the outlying tissue. There did not appear to be much cystitis, nor was there apparently any change of structure in the vicinity of the neck of the bladder, a fact remarkable considering the characteristic tenesmus which always had been a prominent feature of the case, However, a more careful, perhaps microscopical, examination of the parts might have shown substantial alteration. There was marked pelvic peritonitis, in excess overlying and radiating from the base of the ulcer; there were no sloughs in the cavity of the ulcer which seemed to have formed gradually by molecular disintegration, and the mucosa appeared to be of normal character almost to its circumferential edge; in short, the ulcerating cavity seemed as though just recently punched out except over its very base, where a large amount of peritoneal infiltrate had occurred.

I have seen the more or less chronic perforating ulcer at points in the intestinal tract, several times in the duodenum, and I have operated on patients with gangrenous perforating ulcer of the appendix which was neither inflammatory, tuberculous nor typhoidal—a disease which recently by English surgeons, from its course and pathology, has been separated from the ordinary forms of appendicitis. In all these examples I am impressed with a similarity in appearance of the ulceration, the same fundamental cause being common possibly to the entire class although, of course, this view may be questioned. On account of the oftentimes insidious origin and progress leading to fatal termination, these ulcers are of great interest to the physician and surgeon. Particularly would I cite the gangrenous perforating ulcer of the appendix just mentioned, for here death may ensue upon a fatal perforation of the organ which had been preceded by little or no symptoms to indicate the disease, and this is what might be expected in such cases. Much depends upon the location of an ulcer in the body of the appendix and whether or not inflammation has radiated towards the base of the organ and involved the peritoneal attachment, for a large portion of the appendix is devoid of sensory nerves and thus may be involved in disease without giving rise to pain, either objective or subjective. The gangrenous perforating ulcer of the appendix referred to is seldom, if ever, attended by inflammation, and, therefore, may perforate unrestrained by contiguous peritonitis, a feature so conspicuous in the case of the perforating ulcer of the bladder described above. Should the ulcer be situated near the base of the appendix, definite symptoms may exist that will direct attention to disease which may exist at this point.

Mr. Tait did not venture upon an explanation of the pathological cause of these perforating ulcers of the bladder; he simply stated that he endorsed the views of Rokitansky which, unfortunately, he omitted to give in his article. The explanation put forward by Dr. Walker is a generalization too narrow to enlighten us as to the real cause of this malady. He says it is "due probably to local disturbance in, or complete blocking of the terminal, or by an interference with the trophic nerves." The nervous and circulatory systems provide for the nutritional activity of every part and which, being affected by the primal cause, act as secondary factors in the beginning and progress of most morbid processes. This is now known to be true in the case of gastric ulcer which formerly was thought to be caused directly by a localized thrombosis or embolism, but the primal cause has to do with defective cell metabolism, the origin and nature of which we do not quite understand; it may be that the environing blood plasm of the cells supplies the disturbing agency which initiates their destruction.

Although the anatomical constituents of the hollow viscera are somewhat different—for example, the variety of glandular tissue—nevertheless, an argument may be drawn from this fact—that a common cause lies at the root of these perforating ulcers, so much alike in appearance, history, and termination, and that this cause is abnormal cell metabolism which in some mysterious way has been set up in a particular part of the organism, and brings in its train secondary factors which further contribute to the deranged function and finally to the utter destruction of tissue areas involved in the process.—*N. Y. Med. Record.*

